# Prevalence of Mismatch Repair Deficiency and Its Association with Histopathological Parameters in Endometrial Cancer: A Prospective Cohort Study

**DOI:** 10.3390/ijms27156877

**Published:** 2026-07-31

**Authors:** Emmanouela-Aliki Almperi, Chrysoula Margioula-Siarkou, Aristarchos Almperis, Tibor A. Zwimpfer, Alexandros Daponte, Nikoletta Daponte, Thomas Vrekoussis, Theodora Papamitsou, Konstantinos Dinas, Stamatios Petousis

**Affiliations:** 1 Gynaecologic Oncology Unit, 2nd Department of Obstetrics and Gynaeocology, Aristotle University of Thessaloniki, 54622 Thessaloniki, Greece; 2 Gynecological Cancer Centre, University Hospital Basel, University of Basel, 4031 Basel, Switzerland; 3 Department of Biomedicine, University of Basel, 4031 Basel, Switzerland; 4 Cancer Research, Peter MacCallum Cancer Center, East Melbourne, VIC 3002, Australia; 5 Department of Obstetrics and Gynaecology, Medical School, University of Thessaly, 41500 Larissa, Greece; 6 Laboratory of Human Reproduction, Department of Obstetrics and Gynecology, Medical School, University of Crete, 70013 Heraklion, Greece; 7 Laboratory of Histology-Embryology, Department of Medicine, Faculty of Health Sciences, Aristotle University of Thessaloniki, 54124 Thessaloniki, Greece

**Keywords:** endometrial cancer, mismatch repair deficiency, immunohistochemistry, molecular classification, myometrial invasion, histopathology, Lynch syndrome

## Abstract

Mismatch repair deficiency (MMRd) is a critical biomarker in endometrial cancer (EC) for prognostication, Lynch syndrome screening, and immunotherapy eligibility. This study aimed to determine the prevalence of MMRd and its correlation with clinicopathological characteristics in EC. This prospective observational cohort included 93 patients with EC undergoing primary surgical treatment, managed as per the European Society of Gynaecological Oncology (ESGO) guidelines. *MLH1*, *PMS2*, *MSH2*, and *MSH6* expression was assessed through immunohistochemistry (IHC) on formalin-fixed, paraffin-embedded hysterectomy specimens. Associations between mismatch repair (MMR) status and histology, grade, lymphovascular space invasion (LVSI), myometrial invasion, FIGO 2023 stage, nodal status, recurrence, and survival were analyzed. The median age was 66 years. Most tumors were endometrioid (86%), 29% were grade 3, 45.2% showed deep myometrial invasion, and 23.7% had substantial LVSI. MMRd was identified in 41.9% of cases, with MLH1 and PMS2 loss being most frequent, and was significantly associated with endometrioid histology (*p* = 0.027) and deep myometrial invasion (*p* = 0.011). No significant correlations were found with grade, LVSI, FIGO stage, nodal involvement, recurrence, or overall survival. Routine MMR assessment may refine risk stratification and guide individualized treatment decisions.

## 1. Introduction

Endometrial cancer (EC) stands as the most prevalent malignancy of the female reproductive system in developed countries, with a steady incidence rising by ~1–2% annually [1]. Since the 2013 The Cancer Genome Atlas (TCGA) publication defining the genomic landscape of endometrial carcinoma, EC classification and management have shifted toward a molecular framework [2], now embedded in the ESGO/ESTRO/ESP guidelines to refine recurrence risk and overall survival outcomes. The updated clinical approach increasingly incorporates the detection of mismatch repair deficiency (MMRd) or microsatellite instability (MSI) in preoperative biopsies, using immunohistochemistry (IHC) to evaluate the loss of MMR proteins [3], with MMRd reported in up to ~30% of ECs [4]. MMRd reflects the loss of function of MLH1, PMS2, MSH2, or MSH6, resulting in the accumulation of insertions and deletion errors at microsatellites, MSI, and a high tumor mutational burden [5]. The majority of MMRd ECs, approximately 80–90%, are attributed to somatic *MLH1* promoter hypermethylation, causing the epigenetic silencing of *MLH1* [6,7], whereas a smaller subset (~10%) arises in the context of Lynch syndrome, a hereditary cancer predisposition caused by germline pathogenic variants in MMR genes or, less commonly, *EPCAM*, leading to the secondary epigenetic inactivation of *MSH2* [8,9]. The assessment of MMR status for endometrial cancer patients is of major clinical significance. The potential identification of MMRd may indicate the need for genetic testing in order to identify Lynch syndrome in the patient, as well as further familial carriers of relative mutations. Secondly, endometrial cancer patients with MMRd may represent candidates for immunotherapy in advanced stages and in future recurrences, as well receiving both pembrolizumab and levatinib, based on the results of recently published RCTs [10]. However, the prognostic significance of MMRd on its own in endometrial cancer patients remains controversial. Even if some studies have demonstrated improved survival outcomes among patients harboring MMR-deficient tumors, conflicting data exist in the literature, and a definitive consensus has yet to emerge. Furthermore, the correlation of MMRd with various clinical and histopathological characteristics has not yet been clarified, highlighting an area for further research, especially in the context of the increasing but still recent implementation of molecular profiling in endometrial cancer [11]. The main objective of the present study is to assess the rates of MMRd in endometrial cancer patients, as well as define potential correlations with clinical and histopathological characteristics.

## 2. Results

### 2.1. Cohort Characteristics

A total of 93 patients were included in this study. The median age of the patients was 66 years (range, 35–86 years). The most common stage was FIGO IA2, which was observed in 29/93 (31.1%) patients, followed by FIGO IB in 15/93 (16.1%) and FIGO IIC in 14/93 (15.05%). The most common tumor type was the endometrioid type, which was observed in 80/93 (86.0%) patients, whereas a high grade (grade 3) was present in 27/93 (29.0%) endometrial cancer patients. Deep myometrial invasion (≥50% myometrial depth) was detected in 42/93 (45.2%), while substantial lymphovascular space invasion (LVSI) was detected in 22/93 (23.7%) of the study participants (Table 1). The median follow-up duration was 30 months (range: 3–36 months), during which 6/93 patients (6.4%) died (four in the MMRd group and two in the MMRp group) and 2/93 patients (2.2%) experienced disease recurrence (one in each group).

### 2.2. MMRd Rates

MMRd was observed in 39/93 (41.9%) patients in the study group. The MLH1 and PMS2 proteins were the most frequently aberrant, observed in 35/93 (37.6%) and 37/93 (39.8%) patients, respectively, whereas MSH2 loss was observed in 3/93 (3.2%) and MSH6 in 4/93 (4.3%). MMRd was observed in one protein in 1/39 (2.6%) patients, in two proteins in 35/39 (92.2%) patients, in three proteins in 2/39 (5.1%) patients, and in four proteins in 1/39 (2.6%) patients.

### 2.3. Correlation of MMRd with Histopathological Characteristics and Survival Rates

Chi-squared or Fisher’s exact tests were performed to assess the univariate association between MMRd/MMRp status and categorical histopathological parameters (LVSI, deep myometrial invasion, tumor grade, histological subtype, and nodal invasion). Odds ratios (ORs) with 95% confidence intervals (95% CIs) were calculated for each categorical association. The Mann–Whitney U test was used for ordinal FIGO stage data. MMRd status was significantly associated with the histological subtype (*p* = 0.027). Most non-endometrioid tumors were mismatch repair-proficient (MMRp) (11/13, 84.6%), whereas MMRd was more common among endometrioid tumors (37/80, 46.2%). In contrast, MMRd status was not associated with the tumor grade (*p* = 0.939). No significant correlation was observed between MMRd and LVSI (*p* = 0.453), with LVSI present in 11/39 MMRd cases (28.2%), compared with 11/58 MMRp cases (20.3%). MMRd status was, however, significantly associated with deep myometrial invasion (*p* = 0.011), which was more frequent in MMRd tumors (26/39, 66.6%) than in MMRp tumors (16/54, 29.6%). Finally, MMRd showed no association with stage (*p* = 0.987) as the stage distributions were comparable between the MMRd and MMRp groups (MMRd: stage I: 48.7%, stage ΙΙ: 33.3%, stage ΙΙΙ 10.3%, stage IV 7.7% vs. MMR-proficient: 50%, 40.7%, 3.7%, 5.6%, respectively). No significant correlation was observed with nodal invasion (*p* = 1.000) or recurrence (*n* = 2 events, *p* = 1.000). However, a suggestive trend was observed toward an increased overall mortality risk in MMRd patients (*n* = 6 events, 10.5% in MMRd cases vs. 1.8% in MMRp cases, crude OR 2.97 [95% CI 0.52–17.11]). Multivariate logistic regression was performed, including all histopathological variables and the FIGO stage categorized as dummy variables (stage I as a reference). Two variables remained independently associated with MMRd status, namely deep myometrial invasion (OR = 4.30, 95% CI 1.63–11.37, *p* = 0.003) and the histological subtype (OR = 0.04, 95% CI 0.002–0.97, *p* = 0.048), indicating that deeper myometrial invasion increased the odds of MMRd, whereas non-endometrioid histology substantially reduced the likelihood of MMRd. The univariate regression between MMR deficiency rates and various histopathological and survival characteristics is presented in Table 2 and Table 3.

A Kaplan–Meier survival analysis stratified by MMR status showed no significant difference in overall survival (OS) between MMRd and MMRp patients (log-rank test, *p* = 0.262). Similarly, the Kaplan–Meier curves for disease-free survival (DFS) did not differ significantly by MMR status (log-rank test, *p* = 0.837). A univariable Cox regression analysis including hazard ratios (HRs) demonstrated no significant association between MMRd status and OS (HR = 2.57, 95% CI 0.47–14.06, *p* = 0.275, six deaths among 93 patients) or DFS (HR = 1.33, 95% CI 0.08–21.34, *p* = 0.838, two recurrences among 93 patients), although it showed a non-significant trend toward an increased mortality risk in MMRd patients. A fully adjusted multivariable model including additional histopathological covariates was not reliable given the small number of events.

**Figure 1:**
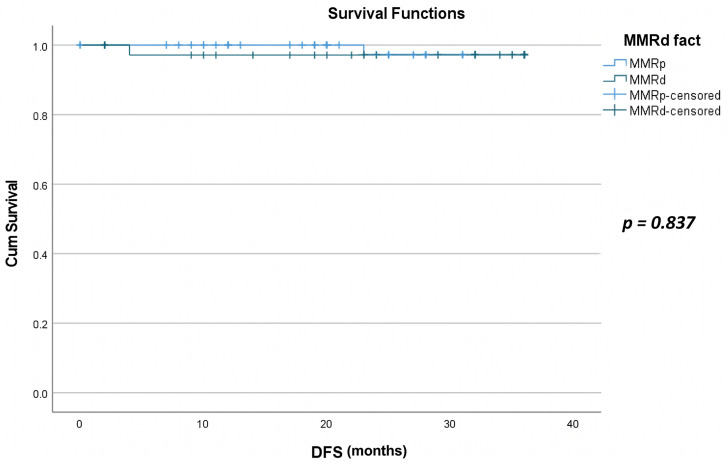
Disease-free survival plots for MMRd and MMRp patients.

**Figure 2:**
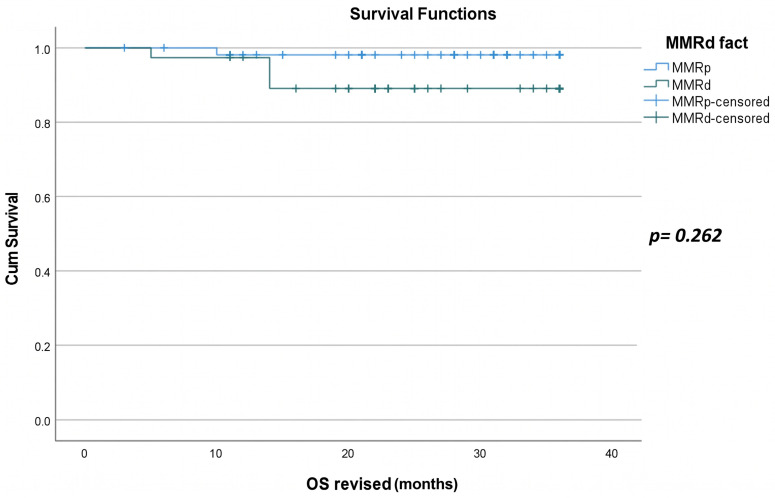
Overall survival plots for MMRd and MMRp patients.

The overlapping survival curves and non-significant log-rank *p*-values indicate that MMRd status, as a single variable, is not associated with differential OS and DFS outcomes in this endometrial cancer cohort. These findings remain exploratory given the limited event count and warrant validation in larger studies with longer follow-up. The Kaplan–Meier survival curves for OS and DFS, stratified by MMR status, are presented below, with time expressed in months (Figure 1 and Figure 2).

## 3. Discussion

The present study reports two main conclusions. First, the overall rate of MMRd was approximately 41.9%, representing one of the highest rates reported in the published literature. Second, MMRd was found to be significantly correlated with the endometrioid histological subtype and deep myometrial invasion, while no significant correlations were demonstrated with other histopathological parameters. The mismatch repair (MMR) system aims to identify and repair replication-associated DNA errors in order to preserve genomic fidelity and integrity during DNA replication [12]. This study sought to determine the prevalence of MMRd in tumor cells among patients diagnosed with endometrial cancer and to evaluate its potential correlations with significant histopathological parameters. In our cohort, 41.9% of patients were observed to have MMRd. The most frequently observed losses of MMR protein expression involved MLH1 and PMS2, occurring in 37.6% and 39.8% of patients, respectively, while only one patient demonstrated the loss of all four MMR proteins. These rates are consistent with those reported in the existing literature. Indeed, in a cohort study by Kim et al., MMRd status was identified in up to 27% of cases, with MLH1/PMS2 loss being the most frequent pattern (68.2%) [13]. Similarly, in a recent study by Aytekin et al., MMRd was detected in 35.1% of cases, with the most common losses involving MLH1 (28.8%) and PMS2 (27.9%) [14]. In another cohort study by Wang et al., the prevalence of MMRd was 25.2%, with MLH1/PMS2 loss observed in 59.5% of cases [15], while Kato et al. reported an MMRd prevalence of 40%, again with MLH1/PMS2 co-loss being the most frequent pattern [16]. The overall prognosis of MMRd ECs falls within an intermediate category. Compared with DNA polymerase epsilon (*POLE*)-mutant ECs, MMRd ECs appear to be more influenced by clinicopathological variables, although not to the same extent as no specific molecular profile (NSMP) ECs [17]. The association between MMRd status and clinicopathological characteristics, as well as the prognostic significance of these factors in MMRd ECs, remains unclear and controversial. For example, while deep myometrial invasion and LVSI significantly worsen the prognosis of MMRd ECs, the tumor grade appears to lack independent prognostic value [18]. The histotype may also lack prognostic significance in this molecular subgroup. Instead, MMRd has been consistently associated with an intermediate prognosis across different histotypes, resulting in poorer outcomes in early-stage, low-grade ECs and improved outcomes in non-endometrioid ECs [19,20]. In the present study, MMRd status was found to be significantly correlated with the histological type and deep myometrial invasion. In contrast, Kim et al. demonstrated that MMRd status was significantly associated with adverse histopathological features such as grade 3 histology (25.0% vs. 11.9%, *p* = 0.040) and LVSI (54.8% vs. 25.0%, *p* = 0.001), with LVSI remaining independently associated with MMRd in a multivariable analysis (RR 3.435, 95% CI 1.447–8.157, *p* = 0.005) [13]. Similarly, in the cohort study by de Freitas et al., MMRd showed statistically significant correlation with two histopathological parameters: endometrioid histology was more frequent in MMRd tumors (87.9% vs. 75.5%, *p* = 0.008), and LVSI was more common, with multifocal LVSI present in 27.6% versus 16.9% of MMRp tumors (*p* = 0.025) [21]. Wang et al. also reported a statistically significant association between MMRd and a higher tumor grade, with grade 3 tumors being more frequent in MMRd compared with MMRp cases (40.5% vs. 23.3%, *p* = 0.004) [15]. Conversely, Kato et al. demonstrated significant correlations between MMRd and several histopathological parameters, including histology (endometrioid vs. non-endometrioid, *p* = 0.01), FIGO stage (I/II vs. III/IV, *p* = 0.03), and histological grade (grade 1/2 vs. grade 3, *p* = 0.01) [16]. Lastly, in the retrospective cohort study by Aytekin et al., no statistically significant associations were found between MMRd and histopathology, FIGO 2009 stage, the depth of myometrial invasion, or LVSI [14]. The correlation that we found between MMRd and myometrial invasion likely stems from a few different interconnected biological factors. MMRd tumors have a significantly higher mutation burden compared to MMRp ones [2]. This is known to increase the neoantigen load and trigger stronger immune cell infiltration within the tumor microenvironment. This elevated immunological activation may lead to a better response to immunotherapy. However, it can also promote local tissue invasion through the increased production of pro-inflammatory cytokines and tumor-associated macrophages [10]. Additionally, MLH1/PMS2 loss, which was predominant in our cohort, typically results from epigenetic silencing via *MLH1* promoter methylation. This characteristic is associated with more aggressive clinicopathological features, including deep myometrial invasion, compared with MSH2/MSH6-deficient tumors [6,21]. The accumulation also of mutations in microsatellites within genes encoding cell adhesion molecules and tissue boundary regulators disrupts cellular signaling pathways that are critical for maintaining tissue boundaries [12]. Moreover, MMRd tumors tend to display significantly higher levels of pro-angiogenic factors—most notably *VEGF* and its downstream signaling. This cascade increases the vascular density and helps tumor cells to invade by remodeling the extracellular matrix and making blood vessels more permeable [10,22]. MMRd status shows a strong histotype correlation with endometrial carcinoma, being more prevalent in endometrioid tumors. Across large series, endometrioid endometrial carcinoma exhibited MMRd in approximately 25–35% of cases [23], with enrichment in higher-grade tumors—reported in about 39.7% of high-grade versus 24.7% of low-grade endometrioid carcinomas [24]. MMRd is also common in undifferentiated and dedifferentiated carcinomas, with some of the highest reported rates (~40–50%) [25], as well as in mixed ECs containing an endometrioid component (16–66%) [26]. Conversely, its prevalence is lower in clear cell carcinoma (~5–15%, depending on the cohort and testing methodology) [27] and carcinosarcomas (~5–20%), often reflecting an endometrioid-like epithelial component [28], and is rare in serous endometrial carcinoma, which is most commonly p53-abnormal and MMRp. It should be noted that the coexistence of MMRd and a serous morphology raises the possibility of a misclassified endometrioid carcinoma or mixed tumor rather than a true serous endometrial carcinoma [29,30,31].

### 3.1. Limitations

This single-center prospective cohort of 93 patients has inherent constraints. The sample size was relatively small, limiting the statistical power for subgroup analyses. Notably, the limited number of non-endometrioid cases (*n* = 13, 14.0%) restricts our ability to draw meaningful conclusions regarding histologic subtype-specific outcomes. Another limitation is the limited follow-up period (median 30 months, maximum 36 months) and the absence of long-term survival outcomes, reflecting the relatively recent integration of MMRd assessment into routine clinical practice. Most importantly, the small number of clinical events (six deaths, two recurrences) compromised our ability to perform robust multivariable survival model analyses. A fully adjusted multivariable Cox model was statistically unreliable, and the univariable analyses remain exploratory. A specific limitation is that *MLH1* promoter hypermethylation was not assessed in MMRd cases. *MLH1* promoter-hypermethylated endometrial cancer represents a distinct higher-risk subgroup associated with adverse clinical factors such as older age at diagnosis, increased body mass index (BMI), higher rates of LVSI, and an advanced disease stage. The inclusion of this information would improve risk stratification and facilitate more tailored management strategies.

### 3.2. Strengths and Future Directions

Offsetting these limitations, this study offers several methodological and demographic strengths. A notable strength of this study is its prospective design, representing the first such investigation in a Greek population, as well as the relatively large cohort from a single center with defined clinical follow-up, which minimized recall and selection bias compared to retrospective designs. All 93 tumors underwent comprehensive molecular characterization according to the TCGA/ProMisE framework, with uniform assignment to defined molecular categories. Staging was standardized across the entire cohort using the 2023 FIGO classification, with cases enrolled before 2023 retrospectively restaged by multidisciplinary tumor board review. Additionally, all surgical procedures were performed by trained gynecologic oncologists, and all specimens were evaluated by dedicated gynecologic pathologists, enhancing the procedural consistency and diagnostic reliability. Furthermore, the study employed a rigorous statistical methodology, with appropriate tests for categorical (χ^2^/Fisher’s exact), ordinal (Mann–Whitney U), and survival data (Kaplan–Meier, log-rank, Cox regression); Firth’s penalized logistic regression was used to obtain stable odds ratio estimates when complete separation occurred. Transparency regarding methodological challenges—such as the use of Fisher’s exact test for low cell counts and the reporting of unreliable estimates rather than unstable odds ratios—reflects careful statistical practice. Future multicenter studies with larger, more diverse cohorts and extended follow-up (≥5 years) are warranted to validate these findings and more precisely define the prognostic significance of MMRd in endometrial cancer. The integration of MMRd status with complementary molecular markers, including *POLE* mutation status and *TP53* alterations, in accordance with the TCGA/ProMisE molecular classification, would enable more refined, biology-driven risk stratification. Larger, adequately powered multicenter cohorts would also help to clarify the relationships between MMRd and clinical outcomes such as recurrence and survival—an association that remains underpowered in single-center series such as ours.

## 4. Materials and Methods

### 4.1. Study Design

This was a prospective cohort study that was conducted as part of a PhD project in the Gynecologic Oncology Unit of the 2nd Department of Obstetrics and Gynecology in Ippokrateio Hospital of Thessaloniki from December 2022 to December 2025. The study protocol was reviewed and approved by the Aristotle University Ethics/Bioethics Committee for Human Research (Protocol No. 9/23.07.2024). Study conduct adhered to Greek legislation (Act 2071/1992, NHS) established by the National Council of Medical Ethics and Deontology and its guidance for observational research.

### 4.2. Patient Recruitment and Inclusion and Exclusion Criteria

Patients with endometrial cancer were recruited, following presurgical clinical and imaging assessment, with histologic confirmation obtained by endometrial biopsy via hysteroscopy. All patients underwent surgical treatment in the Gynecologic Oncology Unit of our department. The exclusion criteria included a history of any another malignancy (gynecologic or non-gynecologic), an inability to undergo surgery due to comorbidities, prior radiotherapy or chemotherapy, and prior surgical procedures involving organs of the female reproductive tract for any reason. All participating patients provided written informed consent after receiving a clear explanation of the study’s objectives and procedures. Staging was performed according to the 2023 International Federation of Gynecology and Obstetrics (FIGO) classification system. Cases enrolled prior to the implementation of the 2023 FIGO system were initially staged under the previous classification and subsequently restaged uniformly according to the 2023 FIGO criteria by the multidisciplinary tumor board, ensuring consistent staging across the entire cohort. As the Gynecologic Oncology Unit of our department represents a European Society of Gynaecological Oncology (ESGO)-accredited department for gynecologic oncology, all cases treated are discussed pre- and postoperatively by our multidisciplinary tumor board (MTB), with the copresence of gynecologic oncologists, radiation oncologists, medical oncologists, pathologists, and radiologists. Postsurgical uterus specimens were collected from 93 female patients, diagnosed with endometrial cancer and surgically treated between the years 2022 and 2025.

### 4.3. Epidemiological, Clinical, and Histopathological Characteristics of Patients

Epidemiological parameters assessed included age, BMI, race, comorbidities (hypertension, diabetes mellitus, renal failure, heart failure), and obstetric history (parity and mode of birth). Presurgical parameters encompassed indications for intervention (vaginal bleeding or endometrial thickness), histologic type and grade, myometrial invasion (evaluation via MRI), FIGO stage, and risk group (low, intermediate, intermediate–high, high). Histological grade was assessed according to the WHO/FIGO criteria and categorized as grade 1, grade 2 (low-grade), and grade 3 (high-grade). Postsurgical parameters included histologic type and grade, myometrial invasion, LVSI, nodal status based on surgical staging, final FIGO stage, and final allocation to a risk group.

### 4.4. Primary and Secondary Outcomes

The primary outcome of the study was the proportion of tumor specimens exhibiting negative expression of at least one of the MLH1, MSH2, MSH6, and PMS2 proteins, enabling the determination of a deficient MMR status in the final uterine surgical specimen. Secondary outcomes included the correlations of MMRd with histopathological parameters, namely the histological subtype, grade, myometrial invasion, nodal status, final FIGO staging, and recurrence or death during the follow-up period.

### 4.5. Specimen Processing and Section Preparation

Postoperatively, within 24 h, hysterectomy specimens were fixed in 10% neutral-buffered formalin and processed routinely. Following macroscopic examination, representative tissue sections were sampled and placed in standard tissue cassettes. Then, they underwent processing in an automatic 12-station tissue processor consisting of sequential immersion in formalin, graded ethanol solutions (increasing concentrations for dehydration), xylene (for clearing), and molten paraffin (for infiltration). Afterwards, tissue blocks were embedded in paraffin and sectioned at a 2.5 μm thickness by a microtome and then were mounted onto positively charged glass slides to enhance tissue adhesion. Then, they were baked in an oven at 65 °C for a minimum of 2 h to ensure complete adherence and remove excess paraffin.

### 4.6. Immunohistochemical Staining Protocol

Immunohistochemical analysis was performed using a fully automated LEICA BOND platform equipped with a two-step, biotin-free, polymer-based detection system (BOND POLYMER REFINE DETECTION, Buffalo Grove, IL, USA). Sections were deparaffinized and antigen retrieval was accomplished through a dual-phase protocol: enzymatic digestion with pepsin for 10 min at room temperature, followed by heat-induced epitope retrieval using an EDTA buffer solution (pH 8.0, BOND EPITOPE RETRIEVAL ER2, Newcastle Upon Tyne, UK) at 99 °C for 20 min. Ready-to-use (RTU) monoclonal antibodies against MLH1, MSH2, MSH6, and PMS2 were applied sequentially to individual sections. The staining protocol consisted of endogenous peroxidase blocking (5 min), primary antibody incubation (25–30 min at room temperature), three wash cycles, post-primary reagent application (20 min), three wash cycles, polymer reagent incubation (20 min), three wash cycles, DAB chromogen development (10 min for visualization), three final wash cycles, and hematoxylin counterstaining (5 min) for nuclear detail. Following the completion of the automated protocol, sections underwent manual dehydration through ascending alcohol concentrations, clearing in xylene, and coverslipping using a mounting medium to enable microscopic evaluation.

#### 4.6.1. Molecular Subclassification

All tumors underwent comprehensive molecular characterization and were assigned to defined molecular categories according to the TCGA/ProMisE framework using POLE and the p53 status, in addition to MMR immunohistochemical assessment. In the present study, outcomes were analyzed with a focus on MMR deficiency (MMRd vs. MMRp); therefore, molecular subtype-specific clinicopathological correlations for POLE- and p53-defined groups are the subject of a separate manuscript and will be reported separately.

#### 4.6.2. Interpretation of MMR Status

The nuclear expression of each MMR protein was evaluated independently. MMR status was classified as deficient when the loss of nuclear staining was observed in one or more proteins. Given that MMR proteins function as heterodimers (MLH1/PMS2 and MSH2/MSH6), intact expression required >90% tumor cell positivity for all four proteins. Complete loss (<10% staining) or subclonal expression (10–90% positivity, typically involving MSH6 or the MLH1/PMS2 dimer) was interpreted as MMRd. MMRp was defined as the preserved nuclear expression (>90%) of all four proteins [32,33].

### 4.7. Statistical Analysis

The Statistical Package for Social Sciences 20.0 (SPSS 20.0) was used to conduct statistical analyses. Continuous variables were summarized as the mean ± standard deviation or median (min–max), depending on their distribution. Normality was assessed using the Shapiro–Wilk test. Categorical variables were summarized as numbers and percentages. Associations between histopathological characteristics and MMR status (MMRd vs. MMRp) were evaluated using the χ^2^ test or Fisher’s exact test, as appropriate. For ordinal variables such as FIGO stage, the Mann–Whitney U test was employed to assess differences between groups. Univariate and multivariate logistic regression analyses were conducted to evaluate the independent associations of histopathological characteristics with MMR status. Due to complete or quasi-complete separation in the predictor space, Firth’s bias-reduced penalized logistic regression was used to obtain stable and finite OR estimates with 95% CIs. The penalized likelihood-ratio test was used to assess statistical significance in the logistic regression models. For survival analysis, OS and DFS were analyzed using the Kaplan–Meier method, with differences assessed by the log-rank test. Cox proportional hazards regression was performed to evaluate associations between prognostic factors and survival outcomes. The proportional hazards assumption was verified using Schoenfeld residuals. Due to the limited number of events (6 deaths for OS and 2 recurrences for DFS), which precluded adequate event-to-variable ratios, a fully adjusted multivariable Cox regression model including additional histopathological covariates was not reliable. Odds ratios and hazard ratios are reported with 95% confidence intervals. All statistical tests were two-sided, and statistical significance was defined as *p* < 0.05.

## 5. Conclusions

In this prospective cohort, MMRd was identified in 41.9% of endometrial cancer cases, most commonly due to MLH1/PMS2 loss. MMRd was significantly associated with the histological subtype and deep myometrial invasion, but not with the tumor grade or LVSI. Routine MMR assessment may improve risk stratification and guide personalized treatment, including immunotherapy. Further studies with longer follow-up are needed to clarify its prognostic significance.

## Figures and Tables

**Table 1:** Epidemiological, histopathological, and clinical characteristics of 93 patients included in the present study.

Characteristic	Value
Age (Mean ± SD)	65.84 ± 11.32
Histopathological Type, *n*(%)	
Endometrioid	80 (86.0%)
Serous	3 (3.2%)
MMMT	5 (5.4%)
Clear cell	1 (1.1%)
Mixed	4 (4.3%)
Myometrial Invasion, *n*(%)	
≥50%	42 (45.2%)
<50%	51 (54.8%)
LVSI, *n*(%)	
Yes	22 (23.7%)
No	71 (76.3%)
Grade, *n*(%)	
Low (Grade 1, Grade 2)	66 (71.0%)
High (Grade 3)	27 (29.0%)
FIGO Stage, *n*(%)	
I	46 (49.5%)
II	35 (37.7%)
III	6 (6.4%)
IV	6 (6.4%)
MMR Status, *n*(%)	
MMRp	54 (58.0%)
ΜΜRd	39 (41.9%)
Nodal Status, *n*(%)	
No	89 (95.7%)
Yes	4 (4.3%)
Recurrence, *n*(%)	
No	91 (97.8%)
Yes	2 (2.2%)
Death, *n*(%)	
No	87 (93.6%)
Yes	6 (6.4%)

MMMT, malignant mixed Müllerian tumor; LVSI, lymphovascular space invasion; MMR, mismatch repair; MMRp, mismatch repair proficiency; MMRd, mismatch repair deficiency; SD, standard deviation; FIGO, International Federation of Obstetrics and Gynecology.

**Table 2:** Univariate logistic regression analysis between MMR deficiency rates and various histopathological and survival characteristics.

Variable	MMRp (*n* = 54)	MMRd (*n* = 39)	Test	OR (95% CI)	*p*-Value
Non-endometrioid vs. endometrioid histology	11 (20.4%)	2 (5.1%)	Chi-squared	0.09 (0.01–0.62	**0.027**
Myometrial invasion ≥ 50% (vs. <50%)	≥50%16 (29.6%)	26 (66.7%)	Chi-squared	4.75 (1.96–11.52)	**0.011**
High grade (vs. low grade)	15 (27.8%)	12 (30.8%)	Chi-squared	1.16 (0.47–2.85)	0.939
Positive LVSI (vs. negative)	11 (20.4%)	11 (28.2%)	Chi-squared	1.54 (0.59–4.02)	0.453
Any nodal invasion	2 (3.7%)	2 (5.1%)	Fisher’s exact	1.41 (0.19–10.43)	1.000
Stage	I: 27 (50.0%) II: 22 (40.7%) III: 2 (3.7%) IV: 3 (5.6%)	I: 19 (48.7%) II: 13 (33.3%) III: 4 (10.3%) IV: 3 (7.7%)	Mann–Whitney U	1.18 (0.73–1.91) *	0.987
Death	2 (3.7%)	4 (10.3%)	Fisher’s exact	2.97 (0.52–17.11)	0.329
Recurrence	1 (1.9%)	1 (2.6%)	Fisher’s exact	1.40 (0.09–23.0)	1.000

* OR per one-stage increase from univariable logistic regression; LVSI, lymphovascular space invasion; OR, odds ratio; CI, confidence interval.

**Table 3:** Multivariate logistic regression analysis between MMR deficiency rates and various histopathological characteristics.

Variable	OR (Firth)	95% CI	*p*-Value
Positive LVSI (vs. negative)	0.85	0.26–2.78	0.788
Myometrial invasion ≥ 50% (vs. <50%)	4.30	1.63–11.37	0.003
High grade (vs. low grade)	1.52	0.38–6.16	0.556
Non-endometrioid vs. endometrioid histology	0.04	0.002–0.97	0.048
Stage	1.15	0.56–2.34	0.707
Any nodal invasion	8.35	0.21–330.53	0.258

LVSI, lymphovascular space invasion; OR, odds ratio; CI, confidence interval.

## Data Availability

The original contributions presented in this study are included in the article. Further inquiries can be directed to the corresponding author.

## Abbreviations

The following abbreviations are used in this manuscript:

BMI	Body mass index
CI	Confidence interval
EC	Endometrial cancer
ESGO	European Society of Gynaecological Oncology
FIGO	International Federation of Gynecology and Obstetrics
HR	Hazard ratio
IHC	Immunohistochemistry
LVSI	Lymphovascular space invasion
MMR	Mismatch repair
MMRd	Mismatch repair deficiency
MMRp	Mismatch repair proficiency
MSI	Microsatellite instability
MTB	Multidisciplinary tumor board
NSMP	No specific molecular profile
OR	Odds ratio
RTU	Ready-to-use
SPSS	Statistical Package for Social Sciences
